# The Eradication of Tuberculosis and the Means by Which It May Be Accomplished

**Published:** 1915-08

**Authors:** F. C. Smith

**Affiliations:** U. S. Public Health Service


### TEXAS MEDICAL JOURNAL

Dr. F. E. Daniel, Founder. Established July, 1885

MRS. F. E. DANIEL, - Publisher and Managing Editor

Published Monthly.—Subscription, $1.00 a Year.

VOL. XXXI AUSTIN, AUGUST, 1915 No. 2

The publisher is not responsible for views of contributors

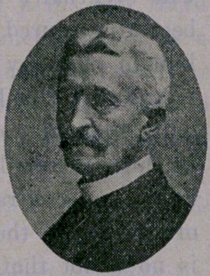

### Original Articles.

### The Eradication of Tuberculosis and the Means by Which It May Be Accomplished.

BY F. C. SMITH, M. D., U. S. PUBLIC HEALTH SERVICE.

If the average physician were asked to state briefly how tuberculosis might be eradicated, the answer would probably be: In one of five ways.

1. By finding a sovereign remedy, a "cure."
2. By producing active specific immunity, by vaccination.
3. By the development of a racial tolerance.
4. By preventing infection through destruction of all tubercle bacilli.
5. By increasing individual resistance through hygienic living.

We are all physicians. We know that there is no sovereign remedy or cure, as the term is commonly used. Most very early cases recover under the dietetic hygienic regimen and most advanced cases fail in spite of remedies. No substance has been found by which it has been satisfactorily demonstrated that the life of a tuberculous guinea pig or other susceptible animal can be saved, and in treating tuberculosis of the skin or mucous membrane no remedy can be applied which is specific. Concerning preventive vaccination, we cannot refrain from a passing tribute to the brilliant scientific work which has been devoted to this subject, but to brutally summarize many years of patient investigation, it must be said that at the present time no practical method of preventing this disease by vaccination, either in man or in animals, has been successfully demonstrated.

The development of racial tolerance is the hope of the defeated, the expectation that if worse comes to worst a degree of immunity will eventually be acquired through the rather hard method of becoming inured to infection. In the tuberculization of the whole human race, say the exponents of this doctrine, will be found our salvation. Drink infected milk, therefore, say they; thank God daily that your child shows a positive cutaneous test, and rejoice in thine tuberculous ancestry. Behold the Jew, oldest of civilized races, and contrast his low mortality from tuberculosis with that of the negro, the Indian and other more primitive men. There is no doubt that there is a deep-seated truth in this theory, but it is of purely academic interest. Nearly two thousand years of exposure to tuberculosis have not resulted in the development of sufficient racial immunity to simplify in any degree the problem of eradicating this disease among the older civilized races.

Prevention of infection is the ideal and doubtless the ultimate goal and is receiving the deserved support of the profession and the laity, but it is found to present great practical difficulties. We have learned the amazing truth that latent infection is practically universal in adults. We know that even the careful consumptive is passing in his urine and feces thousands of tubercle bacilli, and it seems probable that many so-called closed cases do this. We have found that the tubercle bacillus, while easily killed by heat, light and disinfectants, will live for more than a year in water and over one hundred days in the soil, and is very resistant to putrefactive action. There are some who tell of a saprophytic form, of a non-acid fast tubercle bacillus and believe that, like the colon bacillus, it is ubiquitous. We do not believe that the difficulties of eliminating the tubercle bacillus are as great as extremists claim, but it must be admitted that the problem is extremely complex. We know that the matter of dosage is important, that ocasional exposure to infection may be experienced with impunity where gross infection may be fatal, and yet within a few months it has been shown that less than ten tubercle bacilli may cause infection in a child.[1] Just now the world is striving to reduce gross infection by isolation of the terminal cases. Single-minded workers are bravely ignoring lesser sources of infection to provide institutional or other care for the advanced consumptive. With this we are all in hearty accord. Prevent gross infection and we will take chances, if we must, on casual exposure, and yet we are confronted with the great practical difficulty that only a

[1]Webb and Gilbert. Immunity in Tuberculosis. *Journal of the American Medical Association*, September 26, 1914.

small part of the advanced consumptives in this country can yet find needed institutional care. The tremendous, the appalling cost of such a measure has delayed well laid plans and there is hesitancy due perhaps to a suspicion that something else is also necessary. In no part of the world as yet have the means been found to adequately prevent even gross infection. It is the thing to do, but can we do it, or rather, are we willing to pay the price? Not yet, certainly. The world believes that something more is necessary to eliminate tuberculosis than isolation of the advanced cases. And the world is right.

The cultivation of individual immunity through hygienic living is worthy of our support. It is a sociological, not a medical problem. The sanitation of places of employment, the improvement of housing conditions among the poor, the suppression of insanitary occupations, the prevention of child labor and of intemperance, the establishment of a maximum day and perhaps a minimum wage, industrial insurance and pure food laws, all these and many more will be helpful in the eradication of tuberculosis. Powerful forces of society have been loosed to accomplish these things and are receiving the support which the medical profession always accords to such measures. These great reforms should and undoubtedly will come, but at the present time there is another and more urgent matter, purely medical in its aspects, which will presently be urged.

To summarize the present status of the five ordinary ways in which tuberculosis might be eradicated: (1) We have no cure for the cases commonly seeking treatment; (2) we cannot vaccinate; (3) racial immunity is a forlorn hope; (4) prevention must be constantly striven for, but at present, with the yearly advent of thousands of far advanced cases, is not wholly practicable; (5) individual immunity should be developed through hygienic living and will be a great factor in the campaign, but it does not afford the immediate prospect of relief which is so urgently needed. How, then, shall we be saved? By utilizing all that is practicable in the schemes just discussed, by avoiding gross infection, by hygienic living, by treatment of the pretuberculous, and, to make these three possible, *by universal early diagnosis.*

Universal early diagnosis is entirely practicable. It presents no inherent difficulties. There is also nothing new in the suggestion, not even the knowledge that without it all other measures to eradicate tuberculosis, excellent and desirable as they are, will be doomed to failure. The present campaign against tuberculosis is not the aggressive action of an organized army. It is the passive and feeble resistance of an unorganized mob with provision only for its wounded. At present we confidently expect the yearly advent of 150,000 new invalids, advanced cases of tuberculosis.

If we accept modern views there is scarcely a healthy adult who has not already made at least one successful arrestment of early tuberculosis in his own person. Usually these arrestments are made unaided and undiagnosed. A little extra rest, a little letdown from the stress of life, will often do it. If diagnosed early enough arrestment is comparatively simple. How early? Well, from one year to ten years earlier than it is now commonly found. Now does any of you doubt that an expert diagnostician could take any unit of society and if opportunity were afforded forestall the further development of advanced tuberculosis in that unit? This is not conditioned on the segregation of all the advanced cases originally present, although this is greatly to be desired. It is conditioned, of course, on the supposition that when he diagnosed incipient disease, or better the pretuberculous stage, necessary remedial measures would be immediately instituted. Let us make adequate provision for universal early diagnosis, early-enough diagnosis, and tuberculosis as we know it now would be under control in a single generation. It would vanish as an important cause of death with the passing of that pallid multitude which now knocks for admission at our sanatorium doors.

How shall universal early diagnosis be accomplished? In the first place there is no rapid method of diagnosis. No way is known by which clinical tuberculosis may be quickly differentiated from non-clinical, by which that requiring treatment may be known from that which does not. It was at first thought that in the tuberculin tests we had such a method. It would be of incalculable value if we had, because this would enable us to examine large numbers of people in a short time. The Von Pirquet test can be applied by a single operator to several hundred persons daily. But, unfortunately, there is no tuberculin test which can be relied upon to differentiate latent from active tuberculosis. Neither the conjunctival, cutaneous,, percutaneous, nor subcutaneous test will do this, although it may be remarked in passing that the cutaneous test is so delicate as to be of value when found negative and the conjunctival test is not so delicate but that it has some value when found positive. But tuberculin is nothing more than a slight aid to the diagnostician except in detecting disease in the very young. The X-ray is only corroborative in early disease and occasionally helpful in differential diagnosis. The sputum examination should always be made, not once but many times; it is sometimes positive before physical signs appear, but is usually a late phenomenon. No biological method has so far proved reliable in diagnosis. A knowledge of the cellular content of the blood is of but minor assistance and the laborious search of blood, urine and feces for tubercle bacilli is not of great profit and is often misleading. To insure early diagnosis there is nothing, absolutely nothing, that will take the place of a careful history and a thorough physical examination. Moreover, as you of course well know, a chest negative today may six months later show disease, although a chest examined every six months by a competent man could hardly develop disease beyond early incipiency without detection.

To accomplish universal early diagnosis, then, we must have frequent periodical examinations by competent men. Is there any inherent difficulty about that? We have compulsory education, we impose a poll tax upon the voter and an income tax upon the well to do. We publicly demand certificates of health upon various occasions, we vaccinate against smallpox and we take the census, noting with arbitrary precision the age and social condition of each inhabitant. When necessary to protect the State we draft for army or navy, to further the cause of justicee, we impanel on a jury or subpoena as a witness, to protect from cruelty or vice we seize minor children and remove them from harmful environment. Who, then, shall say that we may not require periodical physical examination by the State? Let it become a routine part of school life, but extended considerably beyond the usual school age. Let us exact it from adults as we exact the payment of taxes or the performance of any public duty. It can be done. We are doing harder things than this every day. Some large industrial concerns have already introduced periodical physical examinations among their workmen.[2] The Federal government might well make periodical examinations of all civil service employees. Would it not be practicable for insurance companies to require periodical examinations or at least make a special rate to policyholders who

---

[2]Theodore B. Sachs. The Campaign in Chicago for Medical Examination of Employees. The National Association for the Study and Prevention of Tuberculosis, 1914.

Harry E. Mock. An Efficient System of Medical Examination of Employees. The National Association for the Study and Prevention of Tuberculosis, 1914.

James A. Britton. The Relation of Medical Examination of Employees to the Hygiene of the Working Place and the Efficiency of the Working Force. The National Association for the Study and Prevention of Tuberculosis, 1914.

Knopf. The Modern Warfare Against Tuberculosis. *New York Medical Journal,* October 3, 1914.

Schereschowsky. Physical Examination of Workers. Public Health Reports, November 20, 1914.

complied with the requirement? Frequent periodical examinations could be easily secured. Let us make known the necessity for it. Teach it, preach it, from the cradle through senility. And when early diagnosis is made—early-enough-diagnosis be it always understood—the comparatively simple means required to effect a cure will find ready support from an already thoroughly aroused laity. And the tuberculosis problem will eventually resolve itself into a never-ending search for early cases, always, of course, with the enforcement of those measures already referred to which prevent gross infection and increase individual resistance.

Very early in the evolution of the scheme to insure universal early diagnosis, examinations will probably be relegated to a full-time health officer, who will be an expert diagnostician and will be supplied with all necessary clerical and professional assistance. He will be sufficiently protected by his position from those who may be disgruntled by his diagnosis. He should have authority to require the tuberculous of school age to attend none but the open air schools provided for such, to disbar workers from certain employments, or perhaps from any employment, or to curtail their house of labor, and his mandates might be enforced in the same way that it is proposed to prevent the employment or the excessive employment of children, of pregnant women or of any other class whose health should be safeguarded for the public weal. With the actual treatment of patients he will have nothing to do but will refer them to the family physician. His success will depend entirely on the support given to the measure by the medical profession, which must become sufficiently specialized in the subject to give cordial assent to his diagnosis and to modify certain deeply grounded present day conceptions of the nature of tuberculosis. The ordinary practitioner must be prepared to distinguish between the different types of early disease and advise accordingly. One may need perhaps only a few months rest, or a modification of certain habits of work or play, the abandonment of vicious habits or inordinate amusements, and the adoption of more hygienic practices relative to ventilation, etc. For another, even with early incipient disease, may be required absolute and immediate rest and a ruthless suppression of the most cherished habits and most ardent desires of the individual. To reform these present conceptions and to make these necessary distinctions will require as radical a change in ideas as that caused by the introduction of aseptic surgery, and it will not be surprising if some hesitancy and even opposition is encountered from the more conservative members of the profession.

When frequent periodical physical examinations become routine the examiners will probably always detect disease before rales or other gross manifestations appear. Until that time comes the physician must do his utmost, and more than he has heretofore done, to insure early diagnosis among his clientele. He will not be misled by a tale of malaria, dyspepsia, pleurisy, neuralgia or neurasthenia. He will not sound an alarm merely because the patient is flat chested and emaciated, but he will remember that many robust appearing individuals harbor advanced tuberculosis. He will never neglect the opportunity to make a thorough physical examination of the chest in a warm, quiet room, the patient stripped to the waist and preferably standing, or sitting upon a high stool. He will not rely upon some particular external sign but will employ all the common methods, will stick to one stethoscope, avoid cumbersome and complicated instruments, and will not neglect to listen to the expiration which follows cough. Almost every text-book on physical diagnosis urges auscultation of the inspiration following cough. Monographs have been written on it and yet I am persuaded that few examiners make use of it. We should listen, not to the cough—no one cares how a cough sounds through a stethoscope—not to a forced inspiration which fills your ear with muscle sounds and the grind of costal articulations, but, at the end of a natural expiration, after a slight but evident cough, which forces out the residual air, listen in the natural inspiration immediately following, listen for that still small voice which tells of disease. The physician may, with much credit to himself, refer his patient to a clinical expert in diagnosis, but there is no other way in which he can with honor avoid making an examination which sometimes may not be accomplished at one sitting but requires daily observation of temperature under varying conditions for several weeks. We cannot hurry over an examination or get around it. If we are not willing to spend a half hour or an hour or two hours if necessary to diagnose or exclude tuberculosis we should send the patient to someone who is willing to do so. A busy doctor cannot be expected to spend time on a diagnosis without adequate pay for it, and every city should have publicly paid specialists to examine indigent cases, but there should be on the part of the medical profession such an intelligent grasp of the whole nature of tuberculous infection and such a keen appreciation of the tremendous importance of early diagnosis that no member of the medical profession will ever under any circumstances allow a patient who comes to him for advice to escape a certain diagnosis o rexclusion of active tuberculosis.

